# “I can’t escape my scars, even if I do get better”: A qualitative exploration of how adolescents talk about their self-harm and self-harm scars during cognitive behavioural therapy for depression

**DOI:** 10.1177/13591045241241348

**Published:** 2024-03-22

**Authors:** Anna Kristen, Tanya Lecchi, Maria E Loades, Nick Midgley

**Affiliations:** 1 Division of Psychology and Language Sciences 4919 , University College London and the Anna Freud National Centre for Children and Families, UK; 2 Department of Psychology 1763 , University of British Columbia, Canada; 3 Department of Psychology 1555 , University of Bath, UK

**Keywords:** self-harm, adolescence, discourse, cognitive-behavioural therapy, stigma

## Abstract

Emerging evidence indicates that perceptions of self-harm behaviours and self-harm scars may thwart recovery from depression, yet limited research has explored adolescent accounts of their self-harm and scars during therapy. This study sought to explore how adolescents describe their self-harm behaviours and scars during Cognitive Behavioural Therapy (CBT) and explore the sociocultural discourses that may influence these descriptions. The participants were six female adolescents (aged 14-17 years old) with clinical depression, who were engaging in self-harm. All participants accessed CBT as part of clinical trial evaluating three psychological treatments for major depressive disorder in Child and Adolescent Mental Health Services. Audio-taped CBT sessions were analyzed using discourse analysis. Within CBT sessions, adolescents drew upon stigma discourses in talking about their self-harm. Adolescent also described their self-harm scars as shameful and stigmatizing, and as “proof” of the legitimacy of their depression. It is important for CBT practitioners to understand the context of sociocultural discourses around self-harm behaviours and self-harm scars, which are reflected in how adolescents with depression describe these within therapy and may serve to maintain distress. The study indicates that awareness of use of language and intersecting sociocultural discourses can inform CBT practice.

## Plain language summary

We know that when teenagers are in treatment for depression, perceptions of their self-harming behaviours and self-harm scars can impact recovery. We also know that wider sociocultural beliefs around self-harm tend to be negative and stigmatizing, which might impact how teenagers perceive their self-harm and scars. This study aimed to explore how depressed adolescents talk about their self-harm behaviours and their self-harm scars during therapy for depression. The participants in this study were six female teenagers (aged 14-17 years old) with depression, who were engaging in self-harm. These teenagers participated in a type of therapy called Cognitive-Behavioural Therapy as a part of a larger study evaluating treatments for teenage depression in Child and Adolescent Mental Health Services in the UK. The therapy sessions were recorded, transcribed, and analyzed with a focus on the language used by the teenagers to describe their self-harm and scars. Within the therapy sessions, the teenagers talked about their self-harm in stigmatizing ways, which reflected prominent societal-held beliefs about self-harm. The teenagers in the study also believed that while their self-harm scars were shameful and stigmatizing, the scars also provided a sense of validation that their depression was legitimate. The findings of this study suggest that it could be helpful for therapists to consider how wider sociocultural beliefs around self-harm may impact how teenagers talk about their self-harm and scars in treatment for depression.

## Introduction

Self-harm among adolescents is a major public health concern, given the high prevalence and onset during this developmental period (Geulayov et al., 2018; Muehlenkamp et al., 2019). Large population-based studies in the United Kingdom (UK) have shown marked increases in annual incidence rates of adolescent self-harm over the last decade, especially among female adolescents (Cybulski et al., 2021; Griffin et al., 2018; Patalay & Gage, 2019). In England, an estimated 21,000 adolescents between the ages of 12–17 years old are admitted to the hospital following an episode of self-harm annually, and an estimated 200,000 adolescents each year engage in self-harm but do not access any support via clinical services (Geulayov et al., 2018).

In the public health domain, self-harm is generally recognized as an important target for prevention and intervention efforts, given its robust links with suicide risk (Griep & MacKinnon, 2020). In fact, in 2023 the National Suicide Prevention Strategy for England identified self-harm as a key target for action, calling for improved support for those who engage in self-harming behaviours.

Self-harm has been increasingly understood as a means of expressing or managing distress (e.g., affect-regulation, self-punishment, communication of distress; Taylor et al., 2018). Self-harm encompasses a range of behaviours—including cutting, head-banging, and burning—and those engaging in self-harm often report using multiple methods (Cipriano et al., 2017). Self-harm can be defined as the intentional self-inflicted damage to the body without suicidal intent, and for purposes not socially sanctioned (Klonsky et al., 2014), aligning with the academic consensus of the clinically meaningful differences between non-suicidal self-injury (NSSI) and suicidal self-injury (SSI) (Angelotta, 2015). In the current study, we use the term “self-harm” to best reflect the language used by our research participants.

Self-harm is especially common among adolescents with depression, and particularly those who access help via specialist mental health services, with 18% of adolescents with a diagnosis of major depressive disorder (henceforth referred to as “depression”) reporting self-harm within the past two weeks (Goodyer et al., 2017). Previous studies have shown that co-occurring self-harm behaviours are related to poor outcomes in psychological treatments for depression among adolescents (Abbott et al., 2019; Goodyer et al., 2017). Moreover, emerging evidence indicates that perceptions of self-harm scars may impede treatment, even after self-harming behaviours have stopped (Lewis, 2016). Yet, limited research has investigated adolescent accounts of their self-harm and self-harm scars in the context of psychological treatment for depression. Exploring how adolescents with depression describe their self-harm and their scars during treatment for depression could provide clinically useful information for clinicians working with adolescents who self-harm.

Research has also neglected to consider how wider sociocultural discourses might influence adolescent accounts of their self-harm and self-harm scars, despite the prominence of negative or stigmatizing societal discourses around adolescent self-harm. Over the last two decades, self-harm has come into stark focus in both societal and academic discourses (Cipriano et al., 2017). Discourses can be understood as systems of meaning that construct or create the ways we experience the world (Georgaca & Avdi, 2011). Discourses constitute social realities within a particular historical and sociocultural context; current societal self-harm discourses are thereby socially produced and culturally contingent (Adler & Adler, 2007). Contemporary and western conceptualizations of self-harm are dominated by medical and psychological discourses. For example, drawing upon a medical discourse, self-harm might be described as deviant behaviour, often a pathologized symptom of a mental disorder. Various psychological discourses shape the descriptions of self-harm; for example, the cognitive behavioural model describes self-harm as a maladaptive behavioural response triggered and maintained by patterns of negative thinking, problem-solving deficits, and distress (Slee et al., 2008).

In the UK, there are many prevalent discourses (or rather, perspectives part of a collective societal understanding) surrounding self-harm: For example, stigmatizing discourses may describe self-harm as “attention-seeking,” “manipulative” (Scourfield et al., 2011), and, more generally, attribute mental health problems to individual moral failing (Rössler et al., 2016). Discourses surrounding adolescence that depict the developmental stage as fraught with identity struggles and teenage “drama” likely contribute to propagating talk around self-harm as a “phase,” “fad,” or “performative” (Westers, 2019), or as by-products of identity or subculture such as “goth” or “emo” (Young et al., 2014). Finally, discourses around western beauty ideals permeate discourses around self-harm scars: Self-harm disfigurement can be understood as violating societally constructed beauty standards, thereby eliciting stigma and shame (Kendall et al., 2021; Ngaage & Agius, 2018). These discourses remain pervasive, despite significant research that has shown that the content of self-harm discourses are often misconceptions (Klonsky et al., 2014). These discourses contribute to the stigmatization of self-harm which may be internalized, perpetuate self-harm and mental health problems (Bachtelle & Pepper, 2015), and act as a barrier to help-seeking and recovery (Aguirre Velasco et al., 2020).

Counter-discourses challenging stigmatizing discourses around self-harm engagement have become more prominent in recent years. For example, emerging normalizing discourses depict self-harm as legitimate practice for managing distress (Franzén & Gottzén, 2011), as a sign of strength and resilience for battling mental health problems and describe scarred bodies as beautiful and empowering (Lewis & Mehrabkhani, 2016). Evidently, discourses can play an important role in thoughts and beliefs about ourselves (e.g., scars as a sign of resilience or as disgusting). Given the role of discourses in how we understand and create our “reality and personhood” (Georgaca & Avdi, 2011, p. 148), consideration for how discourses are involved in how adolescents talk about their self-harm may carry implications for therapy, and for addressing self-harm behaviour and resultant thoughts and beliefs that may maintain depression more widely (e.g., negative thoughts about the self).

Few studies have explored how sociocultural discourses surrounding self-harm may shape adolescent accounts of their self-harm and self-harm scars (Arcoverde et al., 2016; Scourfield et al., 2011). One study conducted in Sweden examined how individuals talked about their self-harm online and identified two differing self-harm discourses that shaped how participants described their self-harm and their scars (Franzén & Gottzén, 2011). Normalizing discourses described self-harm as a legitimate practice for managing distress, a reflection of resilience, and described scarred bodies as beautiful. Pathologizing discourses described self-harm as a pathological behaviour, a reflection of weakness in character, and scarred bodies were considered repulsive. Reflecting these dichotomous discourses (i.e., normalizing and pathologizing), another study examining talk online found that some participants perceived their scars as a sign of resilience and reported feeling accepting of them, and others reported their scars were disgusting and shameful and they were not able to accept them (Lewis & Mehrabkhani, 2016). These studies of online communication underscore how discourses are involved in the differing ways that experience and identity is described and understood, and how perceptions of self-harm and scars can have implications for recovery, such that holding more accepting and self-compassionate views of self-harm and scars may facilitate recovery. Yet, few studies have explored how adolescents talk about self-harm and self-harm scars during treatment (Holliday et al., 2020), including Cognitive Behavioural Therapy (CBT).

CBT is the recommended first-line treatment for adolescent depression according to the National Institute for Health and Clinical Excellence (NICE; National Institute for Health and Care Excellence, 2019). The aim of CBT for depression is to change feelings (mood) by changing unhelpful behaviours and thoughts (or changing an individual’s relationship to those thoughts). When self-harm is present, CBT aims to identify and address cognitive, behavioural, and affective factors triggering and maintaining self-harm behaviours. However, research has demonstrated that adolescent self-harming behaviour during CBT for depression is associated with worse outcomes and slower recovery (Gooyder et al., 2017; Wilkinson et al., 2011).

In this context, there is a need to better understand how adolescents describe their self-harm engagement and self-harm scars during CBT sessions. An exploration of first-hand accounts may enhance clinician awareness of how adolescents describe self-harm and scars, and how these descriptions may be influenced by prominent sociocultural discourse about self-harm, which could inform CBT formulation and treatment strategies. The current study therefore aims to explore the sociocultural discourses that adolescents drawn upon in talk about their self-harm and how adolescent various descriptions of their self-harm scars might be situated within such discourses during CBT sessions.

## Methods

### Setting

The current study used data from the Improving Mood with Psychoanalytic and Cognitive Therapies (IMPACT) study (Goodyer et al., 2017), a pragmatic superiority randomised clinical trial investigating whether CBT or short-term psychoanalytic psychotherapy was more effective compared to a brief psychosocial intervention in maintaining the reduction of depressive symptoms at a one-year follow-up. The study recruited 470 participants between 11-17 years old with moderate to severe depression from 15 National Health Service outpatient clinics across three regions in the UK: North London, North-West London, and East Anglia (for the full study protocol see Goodyer et al., 2017). Treatment sessions were audio-recorded as part of the study to monitor treatment fidelity and to support additional analysis of therapy processes.

### Participants

**Figure 1.**
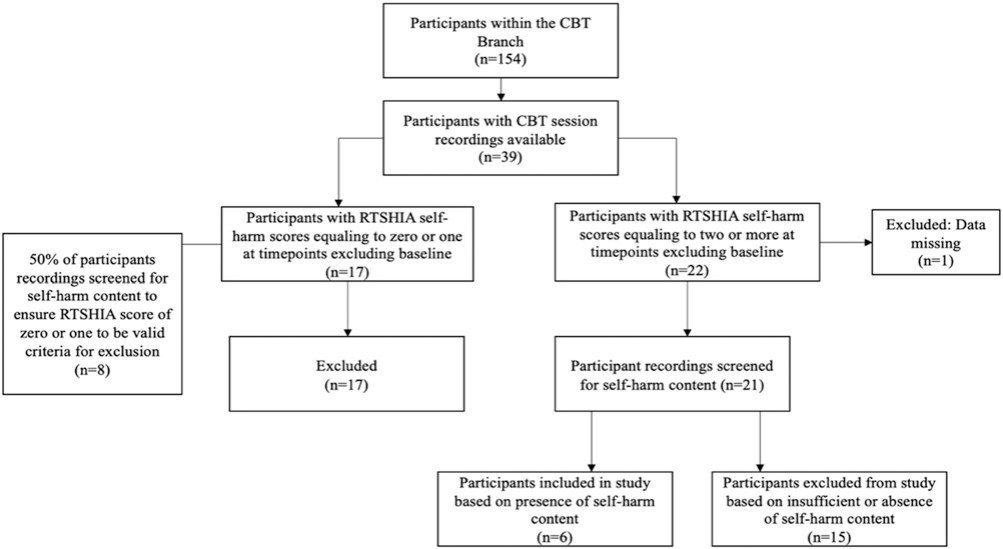
Flowchart of participant selection.

Adolescents participating in the trial who were randomly allocated to the CBT arm (*n* = 154) and had session recordings available were identified as potential participants (*n* = 39). Adolescent scores on the Risk-Taking Self-Harm Inventory for Adolescents (RTSHIA) (Vrouva et al., 2010) was used to identify adolescents who reported engaging in self-harm. See Figure 1 for a flowchart of participant selection. Out of 22 adolescents whose CBT session recordings were reviewed because they had RTSHIA scores of 2 (indicating engagement in self-harm more than once) or above, eight participants had a presence of self-harm content in the recorded sessions. Two of the eight participants’ sessions contained only one brief instance of past self-harming behaviour. These two participants were excluded for lack of sufficient data, and the remaining six participants were included in the study. The participant demographics and the amount of available data for each participant are outlined in Table 1. All names are pseudonyms, and where necessary identifiable material was removed to protect confidentiality.

**Table 1.** Participant Demographics.

Demographic details	Participant pseudonym					
	Emma	Aminah	Morgan	Ellie	Chloe	Jocelyn
Age	17	17	17	16	17	14
Gender	F	F	F	F	F	F
Ethnicity	–	Asian/Asian British	–	Caucasian	–	Caucasian
# Sessions attended	18	20	10	8	6	11
# Recordings available	18	17	10	5	6	7
# Sessions with self-harm data	9	10	4	2	2	4
Range of minutes discussing self-harm per session	2–35	3–26	2–13	5–14	2–12	2–20

*Note.* Baseline data reported. Final row refers to the minimum and maximum number of minutes discussing self-harm within each session for each participant. F = Female; – = Data Missing.

### Procedure

CBT within the IMPACT trial was provided by therapists from a range of disciplines including clinical psychology, nursing, and occupational therapy. Therapists were required to have attended specialist CBT training, either within their core training or post-qualification. Therapists provided treatment within the trial as part of their routine clinical practice in National Health Service Child and Adolescent Mental Health Services. At the outset of the trial, therapists attended a one-day CBT workshop. At this workshop, therapists were introduced to the CBT manual for the trial, which was designed to be used flexibly, and therapists were encouraged to use a personalised, formulation-driven approach to treatment. The manual did not specifically mention self-harm and therapists were encouraged to draw on other cognitive behavioural models when needed. Supervision of CBT therapists was also a part of their routine clinical practice, with no study specific supervision provided. Up to 20 sessions of CBT was available to each adolescent, and parents were involved when needed.

### Ethical considerations

The IMPACT study protocol was approved by Cambridgeshire 2 Research Ethics Committee, Addenbrooke’s Hospital Cambridge, UK (Reference: 09/H0308/137).

### Study design

#### Epistemology

Discourse analysis can be broadly defined as the study of language in use (Potter & Wetherell, 1987). Discourse Analysis is a theoretical-informed approach, operating under the epistemological framework that knowledge, meaning, and understanding are socially constructed through interactions within the social world (see Berger & Luckmann, 1967). Discourse analysis thus assumes that language is context-bound and functional: Language is *used* to construct or create social realities in relation to the immediate context (e.g., therapy) of an interaction and available sociocultural discourses (e.g., self-harm as pathological) (Taylor, 2001). Where other qualitative methods (e.g., thematic analysis) typically focus on what participants talk *about*, discourse analysis is interested in *how* participants talk about a construct of interest (e.g., self-harm) and *why* they might talk about it in a particular way in a particular context. In other words, discourse analysis is interested in “language use, not language-users” (Potter & Wetherell, 1987).

#### Data analysis

The data was transcribed and analyzed in accordance with guidelines outlined by Wiggins and Potter (2008). For each session, all self-harm content was transcribed verbatim. Several close readings of the transcripts occured prior to analysis to facilitate data familiarization. The analytic approach was consistent with synthesized versions of Discursive Psychology and Foucauldian Discourse Analysis, particularly the approach outlined by Willig (2008). Foucauldian Discourse Analysis considers the sociocultural availability of discourses and the ways in which discourses influence social practices and construct subjective experience (Willig, 2008). In this study, prominent societal-held discourses circulating the UK during the time of data collection and data analysis surrounding mental health, self-harm, adolescence, and beauty (as described in the introduction) were considered. This synthesized approach additionally draws upon Discursive Psychology (Edwards & Potter, 1992; Potter & Wetherell, 1987), permitting the examination of the way individuals organize and create their accounts, rhetorical strategies used, and how language is used to achieve interpersonal objectives.

The current study focused upon three analytic levels: (1) construction of discursive objects, i.e. considering how the same discursive object (e.g., self-harm) is constructed in different ways; (2) the sociocultural discourses drawn upon through language, that is considering the wider discourses in which various discursive descriptions are situated within; and (3) the interpersonal function of language, this stage is concerned with the interpersonal function of the description of discursive objects in one particular way at one particular time.

### Sample size

In this study, we determined that the sample size of six participants was suitable for the selected analytic approach. In qualitative research generally, an appropriate sample size is more concerned with the “ability of data to provide a rich and nuanced account of the phenomenon studied,” than the number of participants (Hennink & Kaiser, 2022). In discourse analysis specifically, given that the interest is in the use of language in individual instances of talk, an appropriate sample size is dependant on the availability of text (i.e., individual instances of talk about self-harm and scars) rather than the number of participants.

### Reflexivity

The process of reflexivity is a well-established practice within qualitative research (Dodgson, 2019). The first author completed the current study as part of her MSc thesis. During the analytic process, she was completing her practicum at an adolescent inpatient unit which provided regular exposure to adolescents talking about their self-harm and self-harm scars, supporting credibility of the analysis. However, these experiences may have also contributed to preconceptions about the research findings. To mitigate this risk, the second author reviewed sections of the transcripts and analyses throughout the analytic process. Discourse analysis requires the consideration of language within a sociocultural and historical context, and so it should be noted that the first author has been raised and educated in Canada, France, and the UK. Her own sociocultural experiences are intertwined with the conceptualizations of mental health which necessarily informed the research process. The other authors on this paper were involved with planning the initial study design and supervising the research (T.L and N.M) and one is also a cognitive behavioural therapist (M.E.L). Whilst sharing a similar cultural and educational background, they were able to provide a position of critical curiosity in relation to the emerging analysis conducted by the first author.

### Methodological integrity

To demonstrate methodological integrity, Lincoln and Guba’s (1986) four criteria to establish trustworthiness in qualitative research was used: credibility, transferability, dependability, and confirmability. The strategies applied in this study to achieve trustworthiness are outlined in Table 2.

**Table 2.** Trustworthiness Criteria and Applied Strategies.

Trustworthiness criteria	Purpose	Original strategies	Strategies applied
Credibility	Establishing confidence that the results from the perspective of the participants are credible, reasonable, and true	Prolonged engagement with population of study and the participants Triangulation (data cross-checking) Peer debriefing	- Extensive engagement with adolescents who self-harm and experience depression in a therapeutic context -Extensive engagement with data corpus: Audio recordings and transcriptions were repeatedly reviewed - Analyst triangulation: Second author reviewed numerous analyses throughout the analytic process. Findings were reviewed by the co-authors who have extensive clinical experience - Regular peer and supervisor debriefing throughout the entire research process
Transferability	The extent to which the findings can be transferred to other contexts or settings	Collection of thick descriptive data Purposive sampling	- First author listened to and made notes on all the participants’ sessions amounting to over 63 hours of recordings - Detailed notes and reflections on inpatient unit experience were collected with a focus on self-harm - Sample selected based upon self-harm engagement
Dependability	The extent to which the findings could be repeated	Triangulation Audit trail	- Analyst triangulation - Ongoing process notes were collected throughout participant selection, analysis, and result interpretations
Confirmability	Establishing confidence that results would be confirmed or substantiated by other researchers	Triangulation Reflexivity	- Analyst triangulation - Regular reflections upon analysis process and findings. Weekly reflexive journals on inpatient experiences, with a focus on self-harm were completed

## Results

The results section is presented thematically and reports two groupings of results: (a) use of stigma discourses in self-harm talk, and (b) the paradoxical descriptions of self-harm scars. Although the results were derived from all six adolescents’ sessions, we present excerpts that most explicitly illustrate the themes.

### Use of stigma discourses in self-harm talk

This grouping of findings shows that adolescents in the study drew upon stigmatizing discourses when talking about self-harm in their CBT sessions, including moral deficit discourses, when describing their self-harm engagement:

Aminah: Plus, I don’t know it feels good in that aspect physically, but it also feels good like emotionally like mentally like you know (.) it just you know (.) it’s satisfying.

Therapist: satisfying (.) I’m interested in that word. Satisfying because?

Aminah: I suppose it’s because of the whole blaming yourself for everything you know the self-hatred and all of that and it’s just like it’s just a way of sort of you know letting that out (Session 12).

Here, Aminah draws upon a moral deficit discourse through her use of the terms “blaming” and “self-hatred” depicting herself as inherently bad. Aminah’s use of the term “satisfying” suggests a sense of relief for successfully expelling her perceived faults and badness. Aminah’s use of the term “the whole” alludes to a sense of common knowledge that further draws upon these societal-held stigmatizing discourses around depression and self-harm. Furthermore, Aminah uses a three-part list: “whole blaming yourself,” “the self-hatred,” “and all of that.” Three-part lists have been shown to function as a fact-constructing device to create more convincing accounts (Jefferson, 1990). In this case, Aminah may give weight to these stigmatizing discourses to convey the extent of her depression. She additionally uses an extreme case formulation using the term “everything” which works to curtail the search for evidence or the specifics of what exactly she is to blame for, further drawing upon a moral deficit discourse in her talk. At the end of the excerpt, self-harm is described as a way of “letting out” what Aminah could be describing as self-stigma, that may emerge from, and interact with, stigmatizing societal discourses around depression and self-harm (Aggarwal et al., 2021).

These discourses are similarly drawn upon in the following therapy excerpt. Here, Jocelyn is speaking about her friend who often tells Jocelyn that she is “strong-minded”:

Jocelyn: I know what he [friend] means but it’s doesn’t make sense because it’s not true.

Therapist: Okay. So, what does he mean when he says that you’re strong and you got a strong mind?

Jocelyn: Because I’m like still alive and stuff and um and um I’ve tried really hard to not hurt myself but (.) I don’t see it (.) I don’t think I’m strong because I always feel like hurting myself and I did before […]. (Session 9).

Jocelyn first positions her friend as incorrect. She states: “I know what he means” which disclaims any doubt that she had misunderstood her friend, and rather positions her friend as inaccurate through terms: “doesn’t make sense” and “not true.” Doing this then allows Jocelyn to use causal language that equates character strength as mutually exclusive with mental health problems: “I don’t think I’m strong because I always feel like hurting myself.” This language draws upon moral deficit discourses held by the public, and potentially Jocelyn, that depression is a result of weakness or moral failings in character (Rössler, 2016). She further uses the discursive strategy of repetition with “I don’t see it” and “I don’t think” emphasizing the impossibility of her being strong *and* engaging in self-harm; the repetition of the use of “I” followed by subjective terms such as “see it” “think” similarly functions to create a more irrefutable account.

In these illustrative excerpts, the participants draw upon stigmatizing discourse around self-harm and more generally depression or mental health problems. Primarily, the participants draw upon moral deficit discourses that position their engagement in self-harm and their mental health difficulties as weakness or as moral failings.

### Paradoxical descriptions of self-harm scars

Self-harm scars were a central element of talk for adolescents in the study, the adolescents describe self-harm scars in two ways: as shameful and stigmatizing and as validation of suffering.

#### Descriptions of self-harm scars as shameful and stigmatized

Self-harm scars were a prominent aspect of the adolescents’ talk within therapy. Adolescents in the study described their self-harm scars as shameful and stigmatizing and supported these descriptions through drawing upon stigmatizing discourses around self-harm. This is demonstrated in the following excerpt:

Emma: […] My body is scarred and disgusting from all the cuts and things so it’s like I can’t escape it even if I do get better–that’s what I was thinking.

Therapist: So, when you see scars, you think it’s disgusting?

Emma: I think it’s shameful and horrible.

Therapist: What would be the shame of having–

Emma: –because of–I’ve damaged myself and there are poorer people out in the world who get hurt on their body and they don’t want to, and I hurt my body and it’s healthy (Session 17).

Emma uses the terms “disgusting,” “shameful,” and “horrible” to describe her scars; such evocative language explicitly describes her self-harm scars as shameful and stigmatizing. Emma additionally describes her body as “scarred” and “disgusting.” The use of the conjunction “and” equates her scars with disgust. This talk draws upon discourses around societal beauty standards; specifically, westernized societies places value on perfection and disfigurement from self-harm scars violate these ideals and elicit stigma (Ngaage & Agius, 2018). Drawing upon this beauty discourse gives weight to the description of scars as stigmatizing themselves, beyond the stigma associated with self-harm engagement. Emma then describes that if she were to “get better” she would still not be able to “escape” the stigma evoked by her scars, further emphasizing the description of scars as stigmatizing.

Finally, Emma uses the discursive strategy of comparison and contrast (Wiggins, 2017) in two ways. Firstly, in the latter part of the excerpt, Emma’s language “I’ve damaged” positions herself as an active agent in self-harm. She contrasts this language when describing others who do not engage in self-harm with “people who get hurt” positioning the others as passive agents who have harm done to them. This language highlights Emma’s perceived responsibility in self-harm engagement eliciting shame around her self-harm scars. Secondly, Emma compares and contrasts the description of her “healthy body” to a description of other “poorer people” “who get hurt on their body” and in doing so enacts stigma discourses that depict mental health problems as “entitlement,” “ungratefulness,” and “attention-seeking” to further support the depiction of self-harm scars as shameful.

In the following excerpt, Ellie more implicitly depicts self-harm scars as shameful and stigmatizing:

Ellie: and uh yeah then [after cutting] I just get really worried that cause like after I’ve done it, I always get worried that people are going to like to see it and yeah.

Therapist: Okay. So, you always have to then wear like long sleeves and cover them?

Ellie: Yeah. (Session 6).

Ellie’s description of being “worried” that others will “see” her scars and her agreement of the need to “cover” them describe her scars as shameful and stigmatizing. Both the use of the term “really” as prefix to “worried” and the repetition of the term “worried” may function to emphasize Ellie’s concern of interpersonal stigmatization. Ellie further uses distancing terms when speaking about her scars such as “done it” “see it” rather than naming the self-harm or the scars. This language may function as a discursive strategy to give weight to the descriptions of self-harm scars as if they are too shameful, too stigmatized, and almost too taboo to even name. The use of the term “yeah” in isolation when agreeing with the need to “cover” her scars might indicate that elaboration is not necessary in response to societally implied practice of hiding one’s self-harm scars out of shame and fear of stigmatization.

#### Descriptions of self-harm scars as validation

Adolescents in the study also described their scars as validation or proof of their mental health difficulties, both as validation or proof to themselves and others. This is demonstrated in the following therapy excerpt:

Therapist: From what I am hearing, you are saying that you can’t have depression without cutting yourself, right?

Aminah: I’m saying for me like it’s a sort of way to reassure myself I’m not– I wouldn’t say like this applied to everyone who had depression I’m just saying for me it’s–it’s like I need that kind of physical proof thing because my brain has obviously you know failed me so I can’t trust what I’m thinking so it’s better to like to see, if you know what I mean?

(Session 14).

Aminah’s use of the phrases: “way to reassure myself,” “physical proof,” and “better to see” describes her scars as validation to herself of the legitimacy of her mental health problems. In essence, she may depict her scars to make the invisibility of depression become visible. Aminah may draw upon discourses that question the legitimacy of mental health difficulties as a justification for the need for “reassurance” and to support the description of scars as validation of suffering. She uses discursive strategies to fortify her account: the repetitive use of “for me” and the disclaimer “wouldn’t say like this applied to everyone” function to make her description uncontestable. Finally, the use of rhetorical questioning is known to function to strengthen persuasive impact (Frank, 1990).

In the following excerpt, Morgan describes her scars as a validation of the legitimacy of her suffering to others:

Therapist: […] what sort of things have you been thinking about?

Morgan: Well mainly just doing my wrists in again. I usually think that now and again to see if anyone will actually listen to me this time (.) see how many times I can do it before they actually listen and realize that I really do need help (Excerpt from Session 7).

Morgan describes wanting to “see if” those around her will “actually” attend to her “this time.” The use of these terms in conjunction implies that she is typically dismissed, and wrongfully so. The use of the term “realize” indicates that *others* must come to the *factual* conclusion that she does need support for her mental health difficulties. Here, Morgan’s self-harm scars are described as a signal to others or as proof that Morgan requires support. She further uses the terms “really” as a prefix to “need help” which may function to fortify her description and account.

In the first excerpt, the description of self-harm scars in this way serves to provide reassurance to the participant that her mental health difficulties are authentic. In the second excerpt the description of self-harm scars in the same way may serve to communicate or even convince others of the legitimacy of distress.

## Discussion

The current study explored first-hand accounts of the ways adolescents with depression talk about their self-harm behaviours and self-harm scars within CBT sessions. The two main findings are that adolescents in the study used stigma discourses while talking about self-harming behaviours within therapy, and paradoxically, they described self-harm scars as both shameful and stigmatizing and as validation of their distress to themselves and others. Therapist awareness of adolescent use of discourse and descriptions of self-harm behaviours and scars within therapy may support treatment. Given that the results are reflective of a relatively small sample of adolescents, the findings should be interpreted with some caution.

### Use of stigma discourses in self-harm talk

Our findings show that adolescents in our study drew upon moral deficit discourses in talking about self-harm engagement, whereby mental health problems are attributed to individual moral failings or character deficits (Foucault, 1988; Rössler, 2016). Adolescents drew upon these discourses in talk about the self, suggesting an internalization of stigma discourses, or self-stigma, that are reproduced in talk about their self-harm behaviours. This is consistent with previous research that has shown that self-loathing, self-punishment, and negative views of self are often components of adolescent self-harm talk (Stanicke et al., 2018). Corrigan and Rao’s (2012) progressive model of self-stigma describes how (1) An individual with an “undesired condition” is conscious of public stigma around said condition (p. 2); (2) they perceive these negative public-held evaluations to hold truth; (3) they apply such judgements to themselves; (4) these internalised judgements lead to lowered self-esteem and self-efficacy resulting in the “why try” effect in which individuals maintain the belief that they are not capable (Corrigan & Rao, 2012, p. 2). This process is evident in the CBT sessions we analysed; adolescents drew upon societal stigmatizing discourses around mental health and then attributed these negative public-held beliefs to themselves. Additionally, adolescents’ talk in therapy involved a depicted sense of self-hate, low self-esteem, and lack of self-efficacy. Finally, the “why try” effect, which reflects described hopelessness and worthlessness often associated with depression (Midgley et al., 2015), was evident in adolescent talk in CBT sessions in the current study. The application of the self-stigma model to the current study results suggests that adolescents reproduce stigma discourses in their talk about self-harm within CBT sessions, and addressing this more explicitly within therapy, using this model to frame the discussions, could help to break the vicious cycle in which this consequent negative evaluation of self potentially compounds depression, making the likelihood of future self-harm greater.

We did not identify normalising discourses (e.g., self-harm as resilient or scarred bodies as beautiful) within adolescent talk about self-harm and scars in CBT sessions. This could be attributed to the small sample size of our study or to generational effects, given that the data was collected between 2010 and 2014 when discourses about self-harm may have been different than they are currently. Alternatively, such differences may be accounted for by the context in which talk occurs: the therapy context differs to that of talk online in terms of anonymity, speaking to a professional rather than others who self-harm, and the expectation that therapy aims to reduce self-harm. In CBT sessions, there may be a tendency to focus on problematic behaviour, pathologizing self-harm and describe self-harm as a maladaptive behaviour to be ceased. This may particularly be the case when therapy is accessed within the National Health Service, which is inherently driven by a medical model, even if CBT in this study was biopsychosocial in approach. Moreover, in a therapeutic context, adolescents may find an interpersonal need to save face through prefacing that self-harm is a violation of social norms and presenting descriptions of self-harm that align with therapist descriptions. This context, in hindering access to normalizing discourses, could reinforce negative beliefs about self-harm and exacerbate feelings of shame for adolescents. Normalising discourses could also perpetuate self-harm. It may therefore be helpful for therapists to explicitly ask about these discourses, and to encourage adolescents to express and explore the benefits and drawbacks of these discourses as beliefs about self-harm and scars. Where appropriate, more adaptive beliefs could be developed through Socratic dialogue enabling guided discovery.

### Paradoxical descriptions of self-harm scars

The findings show that adolescents in the study described their scars as both stigmatizing and shameful and as validation of their depression and distress both to themselves and others. These opposing descriptions of self-harm scars reflect a prominent paradox in the literature, which highlights the perceived necessity of keeping self-injury scars private and hidden, while scars simultaneously serving as a communicative act (Chandler, 2018). These findings align with prior research that self-harm scars hold salient meaning for those who self-harm (Lewis & Mehrabkhani, 2016). Adolescent descriptions, which informed the shameful and stigmatizing descriptions, drew upon normative discourses around mental health, self-injury, and beauty. These results suggest that these discourses do not merely exist independently from one another, but rather intersect as compounding stigmatizations.

Goffman (1963) states that stigma may ensue from “abominations of the body” (physical deformities), or “blemishes of individual character” (mental illness, imprisonment, addiction) (Goffman, 1963, p. 2). Matthew et al. (2017) argue that physical markings of addiction amplify stigmatization: The “doubling up” of stigma markings can lead to an exacerbation of shame and self-stigmatization, that is the “physical marking (a stigma in itself) points at or advertises character blemish (a second stigma)” (p. 282). Such concept is applicable to self-harm scars: adolescents seem to describe their self-harm scars (physical deformities) as stigmatizing beyond the fact that scars reflect self-harm behaviour (often seen as a symptom of mental illness, one of Goffman’s blemishes of character) and appear to be described as stigmatizing even after cessation.

Adolescents’ descriptions of disgust reactions to their self-harm scars were related to disfigured bodies through implicitly drawing upon beauty discourses. Disfigurement from self-harm scars violates beauty standards, eliciting shame and stigma beyond self-harm behaviour itself and associated stigmatizing discourses. These results may extend Matthew and colleagues’ (2017) concept of “doubling up” contributing that markings of stigma may “double up” in part due to access to additional stigmatizing discourses around beauty that then heighten shame. Previous research supports this notion: studies have shown that although scars resulting from any cause (e.g., accidental injuries, surgery) are stigmatized (Ngaage & Agius, 2018), self-harm scars elicit more stigma from others compared to non-intentional disfigurement and culturally sanctioned disfigurement (e.g., tattoos) (Burke et al., 2019). This study considers the current sociocultural climate in which scarred bodies are not celebrated.

The shameful and stigmatizing descriptions of scars aligns with previous research on accounts of scars with adult populations (Arcoverde et al., 2016; Lewis & Mehrabkhani, 2016). Although previous studies have found that those with self-harm scars additionally describe their self-harm scars as beautiful, as a sign of resilience, and other positive meanings (Chandler, 2014), this was not identified in the current study. As discussed earlier, the lack of these more positive descriptions may be explained by the inaccessibility of normalizing discourses in a therapy context, and shame around self-harm may prevent adolescents from describing self-harm in a positive way. This study underscores the central focus scars take in adolescent talk which is significant given that shame, stigmatization, and self-stigmatization are barriers towards recovery. It may be useful to explore how adolescents feel about their self-harm scars within therapy.

Scars were additionally described by adolescents as validation of the legitimacy of their distress both to themselves and to communicate legitimacy to others. Adolescents described self-harm scars as a “proof” in seeking validation from self and others as to the reality and legitimacy of their mental health problems. This perceived proof utility may be derived in part from stigmatizing discourses around mental health that cast aspersions onto adolescent reality, positioning them as merely “attention-seeking” rather than experiencing legitimate distress.

It is well-established that the legitimacy of mental health diagnoses is more contested than physical illnesses, and this is in part because mental illness is bound up with judgements about morality (Foucault, 1988). Adolescents have reported experiences of loved ones claiming they are “making it up” or acting out for “attention” (Moses, 2010, p. 998). The need for validation of the legitimacy of their distress through self-harm scars may emerge in part as a response to stigmatizing discourses that question the legitimacy of mental health problems. Such discourses may also be inadvertently perpetuated by referral criteria and thresholds to access services that are dependent on risk.

The description of scars as validation indicate scars may serve the functional purpose of combating these discourses. In this way, the desire for validation of mental health problems to the self and others through scars may serve as a maintenance factor for self-harm. Given the prior discussion on Corrigan and Rao’s (2012) progressive model of self-stigma, it is possible that these discourses around mental health difficulties are internalized, and thus there is a need to validate the legitimacy of mental health difficulties to the self. Prior research has suggested that although self-harm scars are stigmatized, more visible symptoms of mental illness tend to attract helpful attention from those in the inner network (Perry, 2011). The exploration of the ways adolescents describe their self-harm scars during therapy could provide insight into the functional and relational purposes scars might serve.

### Clinical implications

CBT therapists could benefit from understanding the ways wider discourses around self-harm permeate adolescent talk in therapy, and by understanding this, could more sensitively and helpfully explore these within the therapy context. This also applies to better understanding how self-harm scars are described and discussed within the wider discourses, better positioning therapists to support adolescents with self-harm scars to explore and address their thoughts and beliefs about these when they are contributing to problem maintenance. Our findings indicate that adolescents hold complex and paradoxical beliefs about self-harming behaviours and scars and facilitating adolescents to articulate these within therapy may be a helpful part of the journey to recovery.

Specifically, given the absence of normalizing discourses around self-harm or positive description of scars within the sessions we included, therapists might consider sensitively introducing the notion of these normalizing discourses, to gain insight into motivation and barriers to recovery. Our findings demonstrate that self-harm scars are paradoxically described as shameful and stigmatizing, and as validating of distress, indicating that therapists may be required to hold the tensions of these opposing descriptions. Therapists could aim to normalize the paradoxical descriptions and avoid inadvertently colluding with shameful descriptions that silence self-harm talk and perpetuate self-harm as a hidden action.

The descriptions of self-harm scars as shameful highlights the clinical significance for the continued therapeutic focus on scars and their meanings to adolescents even after cessation. This is particularly significant given research that has shown higher levels of scar-related shame is associated with higher likelihood of future self-harm and depression (Bachtelle & Pepper, 2015).

### Limitations

The study is limited by the small sample, and by the limited demographic range of the entirely female and older adolescent sample. While the study focused on the context in which talk occurs, the study did not consider the ways therapists talk and introduction of discourses which may have influenced adolescent talk. It would be interesting to examine whether adolescents draw on different discourses of self-harm in the context of other types of therapy. The current study was also a secondary analysis, and the emerging data corpus was limited due to session focus on depression, yet the analysis of therapy sessions permitted the study of naturally occurring talk mitigating some of the social desirability bias that an interview setting might evoke.

## Conclusion and future directions

In summary, the findings from the present study underscore the potential clinical significance of considering the complex and paradoxical descriptions of self-harm behaviours and resultant self-harm scars, and of the therapist understanding of the discourses drawn upon in adolescent talk about self-harm within CBT. Future research should examine adolescent talk longitudinally over the course of CBT to examine how descriptions of self-harm scars shift over time in relation to recovery, and in the context of other presenting problems beyond depression, with adolescents with more diverse demographics. A deeper engagement with the societal discourses which frame how adolescents talk about self-harm within therapy can shed light on their beliefs and experiences, facilitating the opportunity for more effective therapeutic interventions.
